# Comparative analysis of postprandial changes in the lipidome of capillary plasma and dry blood spots of young women

**DOI:** 10.1007/s00394-026-04119-z

**Published:** 2026-10-07

**Authors:** Stephany G. Duarte, Carlos M. Donado-Pestana, Larissa Rodrigues, Marcos Yoshinaga, Adriano B. Chaves-Filho, Sayuri Miyamoto, Jarlei Fiamoncini

**Affiliations:** 1 Department of Food Science and Experimental Nutrition, School of Pharmaceutical Sciences, University of São Paulo, São Paulo SP, Brazil; 2 Department of Biochemistry, Institute of Chemistry, University of São Paulo, São Paulo SP, Brazil; 3 Department of Food Sciences and Experimental Nutrition, School of Pharmaceutica Sciences, University of São Paulo, Av. Prof. Lineu Prestes, 580, Bloco 14, São Paulo, SP CEP 05508-900 Brazil; 4 Laboratory of Biological Chemistry, Institute of Chemistry, University of Campinas, Campinas, SP, Brazil, Campinas SP, Brazil; 5 Center for Metabolism (CoMETA), University Hospital, University of São Paulo, São Paulo SP, Brazil; 6 Brazilian National Institute of Science and Technology for Hepatology 360° (INCT-Hepatology), Belo Horizonte, MG, Brazil, Belo Horizonte MG, Brazil; 7 Centro de Investigaciones Biomédicas (CEINBIO), Facultad de Medicina, Universidad de la República, Montevideo, Uruguay; 8 Programa en Alimentos y Salud Humana (PAyS), Facultad de Medicina. Universidad de la República, Montevideo, Uruguay; 9 PinguisLab, São Paulo SP, Brazil; 10 Cruzeiro do Sul University, São Paulo SP, Brazil; 11 Division of Tumor Metabolism and Microenvironment, German Cancer Research Center (DKFZ) and DKFZ-ZMBH Alliance, Heidelberg, Germany

**Keywords:** Lipidomics, Lipid metabolism, Phospholipids, Absorptive phase, Biomarkers

## Abstract

**Purpose:**

The postprandial period provides a dynamic window to assess metabolic function, as nutrient intake elicits measurable shifts in metabolism, that can be used to assess health status. Traditional venipuncture, though standard for blood sampling, poses practical limitations for postprandial studies that require repeated collections. This study evaluated the feasibility of using dry blood spots (DBS) as a minimally invasive alternative for monitoring postprandial lipemia, which can vary significantly after the intake of high-fat meals.

**Methods:**

Ten young women (26.1 ± 4.5 years) participated in a controlled clinical trial designed to evaluate lipidomic responses to a standardized, energy-dense meal using both DBS and capillary plasma samples. An analytical method based on ultra-high-performance liquid chromatography coupled with high-resolution mass spectrometry (UHPLC-ESI-TOFMS) was employed for the quantitative profiling of lipid species.

**Results:**

The postprandial lipidome derived from DBS and plasma samples exhibited high concordance, with triglycerides (TG) and free fatty acids (FFA) standing out among 18 identified lipid classes as the most responsive to food intake in both matrices. Postprandial TG concentrations increased by 64% in plasma and 34% in DBS, while FFAs decreased by 36% and 14%, respectively. In addition, DBS-specific postprandial lipidomic signatures were observed, including alterations in the levels of glycerophospholipids and sphingolipids, along with a peculiar kinetic pattern of phosphatidylethanolamines levels following food intake.

**Conclusion:**

These findings highlight the suitability of DBS for capturing postprandial lipidomic changes in circulation, particularly in TG and FFA levels, and support its utility as a practical and scalable tool for metabolic phenotyping in nutritional research, population studies, and clinical monitoring.

**Supplementary Information:**

The online version contains supplementary material available at https://doi.org/10.1007/s00394-026-04119-z.

## Introduction

For a proper assessment of the relationship between diet and health, accessible and reliable biomarkers are needed for understanding nutrient-mediated metabolic regulation. The evaluation of postprandial metabolism offers a valuable approach to gaining detailed insights into health status by examining adaptations to environmental cues such as nutrient availability. The postprandial period is marked by transient changes in circulating concentrations of nutrients, metabolic intermediates, and signaling molecules. In contrast to plasma glucose levels, which typically exhibit a transient increase shortly after a meal, circulating levels of triglycerides begin to rise normally after the first hour following food intake. The increased postprandial lipemia can persist for 5–8 h after consuming a standard meal containing 30–60 g of fat [1]. Importantly, elevated postprandial triglycerides show a stronger association with cardiovascular risk [2, 3], highlighting the clinical relevance of investigating postprandial lipid metabolism.

Although lipid research has been conducted for centuries, the field has gained momentum with the emergence of lipidomics, a subfield of metabolomics. Advances in lipid analysis techniques over the past few decades have enabled the simultaneous identification and quantification of hundreds and even thousands lipid metabolites, which are key players of energy metabolism and potent modulators of inflammation [4]. Despite advances in knowledge, our understanding of the role of lipids in postprandial metabolism remains limited. Research has primarily focused on lipid classes, such as triglycerides (TG), free fatty acids (FFA), and lipoprotein particles, rather than isolated lipid species, often overlooking lipid classes like glycerophospholipids and sphingolipids. Previous lipidomics studies have evaluated lipid profiles during the postprandial response in healthy and diabetic individuals, identifying differences in diacylglycerols, triacylglycerols, phosphatidylethanolamines, and phosphatidylinositols [5, 6]. However, the postprandial kinetics of the vast majority of lipid species remain largely unknown. Making this picture even more complex, depending on the lipid class, their postprandial kinetics can display a marked interindividual variability, and are influenced by test meal composition and other environmental factors.

A major limitation in postprandial studies is the need for multiple blood samples to accurately capture the time-resolved metabolic changes that occur after meal consumption. Typically, venipuncture is the preferred method, despite its invasive nature and the need for trained personnel and specialized infrastructure, which collectively increase study costs. As a more practical alternative, dry blood spots (DBS) represent a low-cost, non-invasive method for capillary blood sampling in postprandial studies [7, 8]. In this context, this study aimed to comparatively assess the lipidomic profiles of DBS and capillary plasma samples collected from young women during a high-glucose, high-fat dietary challenge, using ultra-high-performance liquid chromatography (UHPLC) coupled to high-resolution mass spectrometry (ESI-TOFMS).

## Materials and methods

### Participants

The study protocol was approved by the Ethics Committee of the School of Pharmaceutical Sciences (CAAE: 15438019.7.0000.0067). Participants who agreed to participate in the study signed an informed consent form. Ten young women (26.1 ± 4.5 years) were recruited following a screening that excluded individuals with a diagnosis of diabetes, pregnancy, casein allergy, and lactose intolerance. Participants had a mean body weight of 69.3 ± 12.6 kg and a mean height of 1.65 ± 0.08 m, resulting in a mean body mass index (BMI) of 25.6 ± 4.5 kg/m². Average waist and hip circumferences were 77.8 ± 10.2 cm and 100.9 ± 9.6 cm, respectively.

### Dietary challenge and sampling of DBS and capillary plasma

After a 10-hour overnight fast, volunteers underwent a dietary challenge consisting on the ingestion of a mixed liquid meal containing 75 g glucose, 60 g canola oil, and 20 g casein (chocolate flavored), dissolved in water to a final volume of 200 mL. This energy-dense drink was not designed to replicate a typical meal, but it is a well-acknowledged metabolic challenge [9].

Both DBS and plasma samples were obtained from capillary blood collected by finger prick using a sterile lancet, following fingertip disinfection with an isopropyl alcohol swab. This approach enabled a direct comparison between two minimally invasive sampling strategies derived from the same biological source, while reducing analytical variability. Samples were obtained in the fasting state immediately before the test meal (0 min) and at 60, 90, 120, and 150 min after meal intake. These time points were selected to capture the early to mid-postprandial phase, characterized by rapid metabolic changes, particularly in free fatty acids and the initial rise in triglycerides. Plasma was separated from capillary blood collected into 250 µL EDTA-treated microtubes (Microvette, Sarstedt) by centrifugation at 4000 × *g* for 10 min at 4 °C. DBS samples were obtained by applying blood drops directly onto a DBS card (Whatman 903 Protein Saver Filter Paper). After collection, DBS cards were allowed to dry for 2 h at room temperature and then stored at − 80 °C in aluminum sealable bags (Whatman, GE Healthcare Life Sciences) containing a sachet of desiccant material. DBS samples were collected at the same time points as capillary blood.

### Lipidomic analysis

Plasma and DBS samples were subjected to lipid extraction according to the method described by Matyash et al. [10], with modifications. In brief, 20 µL of plasma or two DBS punches (3-mm diameter each) were mixed with 300 µL of cold methanol containing 10 µM butylated hydroxytoluene and 100 µL of an internal standards mixture (Suppl. Table S1). After vortexing for 30 s, 1 mL of methyl tert-butyl ether (MTBE) was added to the samples, which were then kept under constant agitation at 1000 rpm for 1 h at 20 °C. Subsequently, 300 µL of water was added to the mixture, followed by homogenization and incubation in an ice bath for 10 min. After centrifugation at 10,000 *g* for 10 min at 4 °C, the supernatant containing the lipid extract was transferred to a new tube and dried under N_2_. The dried extracts were resuspended in 100 µL of isopropanol. A pooled sample containing 5 µL of each of the samples was prepared for reproducibility analysis. The injection volume was adjusted to 2 µL and 3 µL for positive and negative ionization modes, respectively.

Lipid extracts were analyzed by ultra-high-performance liquid chromatography (UHPLC Nexera, Shimadzu, Kyoto, Japan) coupled to a high-resolution mass spectrometry (ESI-TOFMS) (Triple TOF 6600, Sciex, Concord, USA), as previously described by Chaves-Filho et al. [11]. Samples were loaded onto a Cortecs^®^ (UPLC^®^ C18, 1.6 μm, 2.1 mm i.d. × 100 mm) column at a flow rate of 0.2 mL min^− 1^ and the oven temperature was maintained at 35 °C. For reversed-phase liquid chromatography (RPLC), mobile phase A consisted of water/acetonitrile (60:40), while mobile phase B was composed of isopropanol/acetonitrile/water (88:10:2). Mobile phases A and B were supplemented with ammonium acetate or formic acid (at a final concentration of 10 mM) for negative or positive ionization mode experiments, respectively. The LC gradient went from 40 to 100% phase B over the first 10 min, held at 100% B from 10 to 12 min, decreased from 100 to 40% B from 12 to 13 min, and held at 40% B from 13 to 20 min. Mass spectrometry analysis was performed using Analyst^®^ software (Sciex, version 1.7.1) in positive and negative ionization modes. The scan range was set from 200 to 2000 Da. Data for the identification and quantification of lipid molecular species were obtained using the information-dependent acquisition (IDA) method. The accumulation time for ions in MS1 and MS2 experiments was set to 100 and 20 milliseconds, respectively. A total of 20 potential precursor ions were monitored per cycle, totaling 0.55 s. The applied spray voltage conditions were adjusted to − 4.5 kV and 5.5 kV (for negative and positive modes, respectively) while the cone voltage was set to +/− 80 V. The curtain gas pressure was set to 25 psi, with nebulization and source heating gases set to 45 psi and the interface temperature to 450 °C.

Data obtained from precursor ion fragmentation (MS/MS) were analyzed using PeakView^®^ software, and lipid molecular species were manually identified based on the m/z of the precursor ions and their respective fragments. Lipid annotation was performed manually with the aid of an “in house” built library of precursor-specific fragments ions. Lipid quantification was conducted using MultiQuant^®^ software, where the areas of the precursor ion peaks were normalized to the areas of internal standards. The concentration of lipid species was calculated by multiplying the analyte/internal standard area ratio by the concentration of the corresponding internal standard or by using external calibration curves.

### Statistical analysis

Data analysis was performed using GraphPad Prism v.10 software (GraphPad Software, San Diego, CA, USA). Data normality was evaluated using the Shapiro-Wilk test. Only differences with *p* < 0.05 were considered significant, and the results were expressed as mean ± standard error. To identify lipid species that discriminated sampling time points during the dietary challenge, partial least squares discriminant analysis (PLS-DA) models were constructed based on lipidomic data obtained from capillary plasma and DBS samples, analyzed separately. Variables (lipids) with a variable importance in projection (VIP) value greater than 1 were considered relevant for discriminating between collection times or as variables responding to the meal intake. Mixed-effects model analysis was performed to test for differences between time points during the dietary challenge. Correction for multiple comparisons, controlling the false discovery rate (FDR), was performed using the Benjamini–Hochberg method. Additionally, Pearson correlation analysis was used to evaluate correlations between lipid species.

## Results

**Fig. 1:**
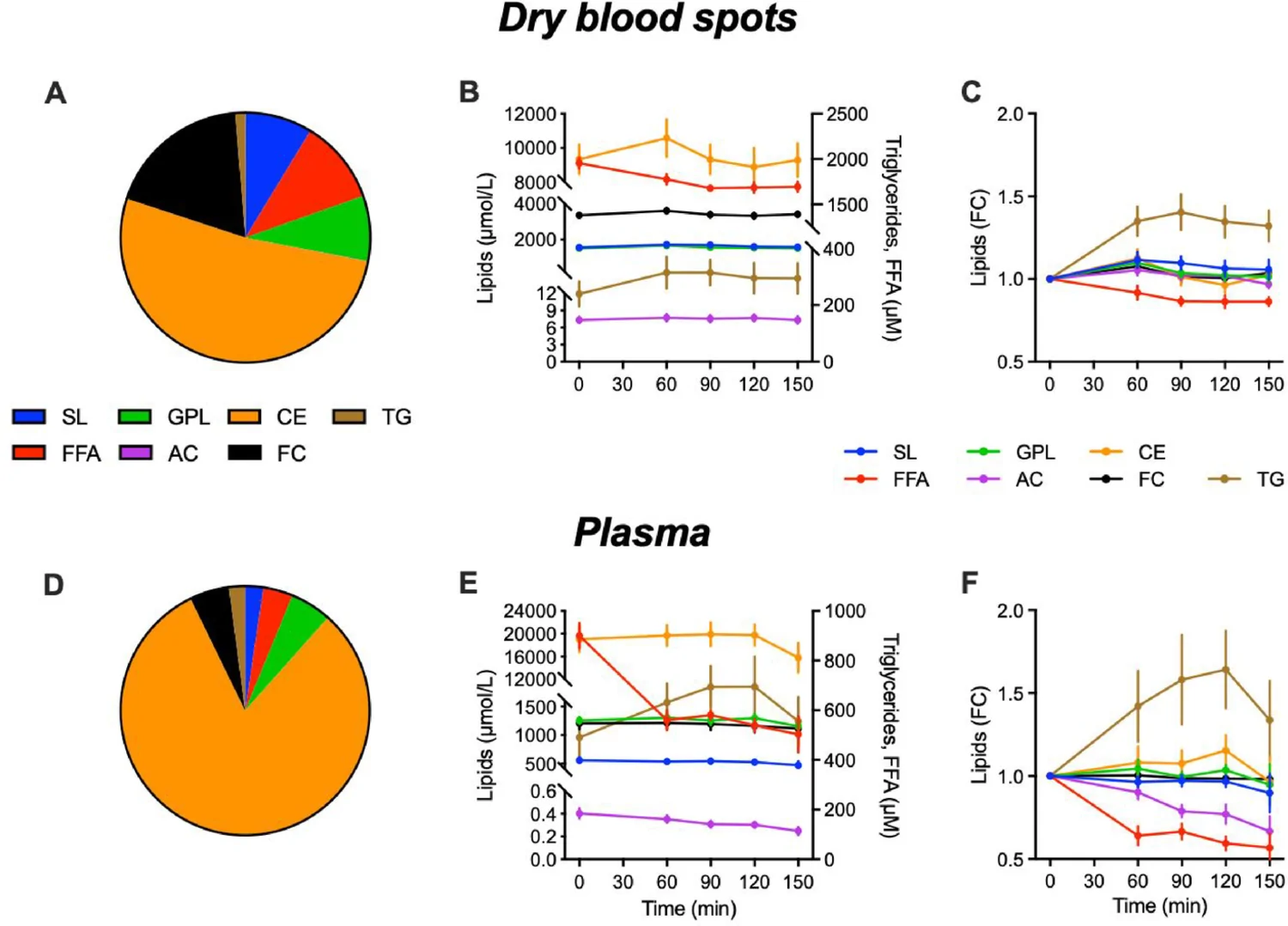
Lipid classes in DBS and plasma during the dietary challenge.** A**—Percentual participation of the most abundant lipid classes in DBS after an overnight fasting. **B**—Concentration of the most abundant lipid classes in DBS during the dietary challenge. **C**—Fold-changes of the levels of most abundant lipid classes in DBS during the dietary challenge. **D**—Percentual participation of the most abundant lipid classes in plasma after an overnight fasting. **E**—Concentration of the most abundant lipid classes in plasma during the dietary challenge. **F**—Fold-changes of the levels of most abundant lipid classes in plasma during the dietary challenge. Data presented as mean ± standard error (*n* = 10)

Lipidomic analysis identified 18 most prominent lipid classes, comprising 338 lipid species in capillary plasma and 306 species in DBS. The lipidomic profiles were comparable between matrices in terms of chemical diversity. In both, triglycerides (TG), phosphatidylcholines (PC), and free fatty acids (FFA) represented the dominant classes (based on the number of quantified metabolites), accounting for approximately half of the identified species (Table 1). Quantitatively, plasma and DBS lipidomes were also comparable, with cholesterol esters (CE) constituting the predominant class, and FFA, glycerophospholipids (GP), free cholesterol (FC), TG, and sphingolipids (SL) also contributing substantially (Fig. 1). These similarities should be interpreted in light of the intrinsic differences between matrices, as DBS represent whole blood and therefore include cellular components (e.g., erythrocytes and leukocytes), which may influence the relative abundance of certain lipid classes compared with capillary plasma. A complementary multi-method comparison further supports these findings, highlighting overall concordance alongside matrix-dependent differences (Suppl. Figure S1).

**Table 1:** Lipid classes in plasma and dry blood spot (DBS) samples, and the corresponding number of molecules quantified in each class

Lipid classes	Abbr.	DBS		Plasma	
		#	%	#	%
Sphingomyelins	SM	32	10.5	33	9.9
Ceramides	Cer	31	10.1	19	5.7
Glucuronosphingolipids	GSL	6	2	0	0
Free Fatty Acids	FFA	35	11.4	37	11.1
Free Cholesterol	FC	1	0.3	1	0.3
Cholesterol Esters	CE	19	6.2	11	3.3
Oxylipins	OL	2	0.7	2	0.6
Lysophosphatidylcholines	LPC	3	1.0	8	2.4
Phosphatidylcholines	PC	44	14.4	50	15
Phosphatidylcholine ether	oPC	6	2.0	0	2.4
Plasmalogen Phosphatidylcholines	pPC	4	1.3	0	3.6
Phosphatidylethanolamines	PE	19	6.2	15	4.5
Phosphatidylethanolamines ether	oPE	0	0.0	1	0.3
Plasmalogen Phosphatidylethanolamines	pPE	15	4.9	14	4.2
Phosphatidylinositols	PI	5	1.6	12	3.6
Phosphatidylserines	PS	4	1.3	0	0.0
Acylcarnitines	AC	8	2.6	7	2.1
Triglycerides	TG	72	23.5	104	31.1
Total		306	100	338	100

Independent PLS-DA models were constructed with data from plasma and DBS samples. Considering all lipid species quantified, the models did not identify major changes in the overall lipidome among the 5 sampling intervals (ANOVA *p*-values of cross-validated residuals for the Y-variable = 0.58 in plasma and 1 in DBS). Scores and loadings plots, as well as the validation plots (500 permutations), are presented in Suppl. Figure S2. Nevertheless, the models identified TG and FFA as the most discriminant variables of the samples collected in different time points (81 lipids had VIP > 1 in plasma and 88 lipids in DBS samples). Of these, 36 discriminant lipid species were shared between the two matrices. Unlike capillary plasma, DBS samples showed several GP (phosphatidylethanolamines (PE) and their plasmalogen derivatives (pPE), as well as phosphatidylserines (PS) alongside sphingolipids, as postprandially modulated lipids (Table 2). Overall, lipid concentrations fluctuated more in capillary plasma than in DBS, as reflected by fold change values in response to meal intake. In both matrices, TG displayed the most pronounced postprandial increase (64% and 34% for plasma and DBS, respectively), whereas FFA showed the most marked decrease (36% and 14%, respectively; Fig. 1).

**Table 2:** Lipid species selected based on VIP scores > 1 from PLS—DA models, representing the most discriminant lipids across postprandial period in plasma, dry blood spots (DBS), or both matrices

Plasma (45)		DBS (52)		Plasma and DBS (36)	
AC 16:0	TG 16:0/ARA/22:4	SM d17:1/24:1	pPE p18/18:1	AC 14:1	TG 18:1/18:2 × 2
AC 16:1	TG 16:1/18:2/18:3	SM d18:0/16:0	pPE p18/18:2	FA 14:0	TG 18:1 × 2/18:2
FA 18:0	TG 16:1/18:2/ARA	Cer d18:1/22:1	pPE p18/22:4	FA 14:1	TG 18:1 × 2/18:3
FA 19:0	TG 16:1/18:2 × 2	GalAc-3 H-cer d18:1/22:1	pPE p18:1/16:0	FA 16:0	TG 18:1 × 2/20:0
FA 19:1	TG 16:1/18:3/ARA	GalAc-3 H-cer d18:1/23:0	pPE p18:1/18:1	FA 16:1	TG 18:1 × 2/20:1
FA 22:3	TG 16:1 × 2/18:2	HODE	pPE p20/ARA	FA 17:0	TG 18:1 × 2/20:2
FA 22:4	TG 16:2/18:1/18:3	FA 18:3 n-3	PS 18:0/20:3	FA 17:1	TG 18:1 × 2/ARA
FA 24:1	TG 16:2/18:2 × 2	oPC o16/18:2	TG 15:0/18:1/18:2	FA 18:1	TG 18:1 × 3
FA 26:1	TG 18:0/18:1/ARA	PC 15:0/16:0	TG 16:0/18:1/18:2	FA 18:2	TG 18:2 × 2/18:3
FA EPA	TG 18:1/18:2/20:2	PC 15:0/18:2	TG 16:0/18:1/18:2-OH_A	FA 20:2 n-6	TG 18:2 × 3
LPC 18:2	TG 18:1/18:2/20:3	PC 18:0/20:2	TG 16:0/18:1/20:3	FA 20:2 n-9	
PC 14:0/DPA	TG 18:1/18:2/22:0	PE 16:0/18:1	TG 16:0/18:1/DHA	FA 20:3 n-6	
PC 18:2/20:2	TG 18:1/18:2/ARA	PE 16:0/18:2	TG 16:0/18:2/20:1	FA 20:3 n-9	
PI 18:0/18:2	TG 18:1/18:3 × 2	PE 16:0/20:1	TG 16:0 × 2/ARA	FA 22:2	
TG 14:0/18:2/18:3	TG 18:1 × 2/22:0	PE 16:0/20:2	TG 16:1/18:1 × 2	FA 24:2	
TG 14:1/16:1/20:3	TG 18:2/18:3/ARA	PE 16:0/20:3	TG 17:0/18:1/18:2	FA ARA	
TG 16:0/16:1/ARA	TG 18:2/18:3 × 2	PE 16:0/ARA	TG 17:1/18:1/18:2	FA DHA	
TG 16:0/16:1/DHA	TG 18:2 × 2/20:3	PE 16:0/DHA	TG 18:0/18:1/18:2-OH	FA DPA n-3	
TG 16:0/16:2/DHA	TG 18:2 × 2/ARA	PE 18:0/18:1	TG 18:1/18:1/18:3-OOH	FA DPA n-6	
TG 16:0/18:0/ARA		PE 18:0/18:2	TG 18:1/18:2/18:2-OOH_A	PE 18:1/18:1	
TG 16:0/18:1/20:2		PE 18:0/ARA	TG 18:1/18:2/18:3-OH	TG 16:0/18:1/20:1	
TG 16:0/18:1/ARA		PE 18:1/DHA	TG 18:1/18:2/18:3-OOH	TG 16:0/18:1/22:4	
TG 16:0/18:1/EPA		PE 18:2/18:2	TG 18:1 × 2/18:2-OH	TG 16:0/18:2/ARA	
TG 16:0/18:2/20:2		pPE p16/18:1	TG 18:1 × 2/18:2-OOH	TG 18:0/18:1 × 2	
TG 16:0/18:2/22:4		pPE p16/20:1	TG 18:1 × 2/18:3-OH	TG 18:1/18:2/18:3	
TG 16:0/18:2/24:1		pPE p16/22:4	TG 18:1 × 2/20:3	TG 18:1/18:2/20:1	

AC, Acylcarnitines; CER, Ceramides; FFA, Free Fatty Acids; GalAc, Glucuronosphingolipids; HODE, Hydroxy-octadecadienoic acid; LPC, Lysophosphatidylcholines; PC, Phosphatidylcholines; PE, Phosphatidylethanolamines; pPE, Plasmalogen phosphatidylethanolamines; PI, Phosphatidylinositols; PS, Phosphatidylserines; oPC, Phosphatidylcholine ether; SM, Sphingomyelins; TG, Triglycerides

**Fig. 2:**
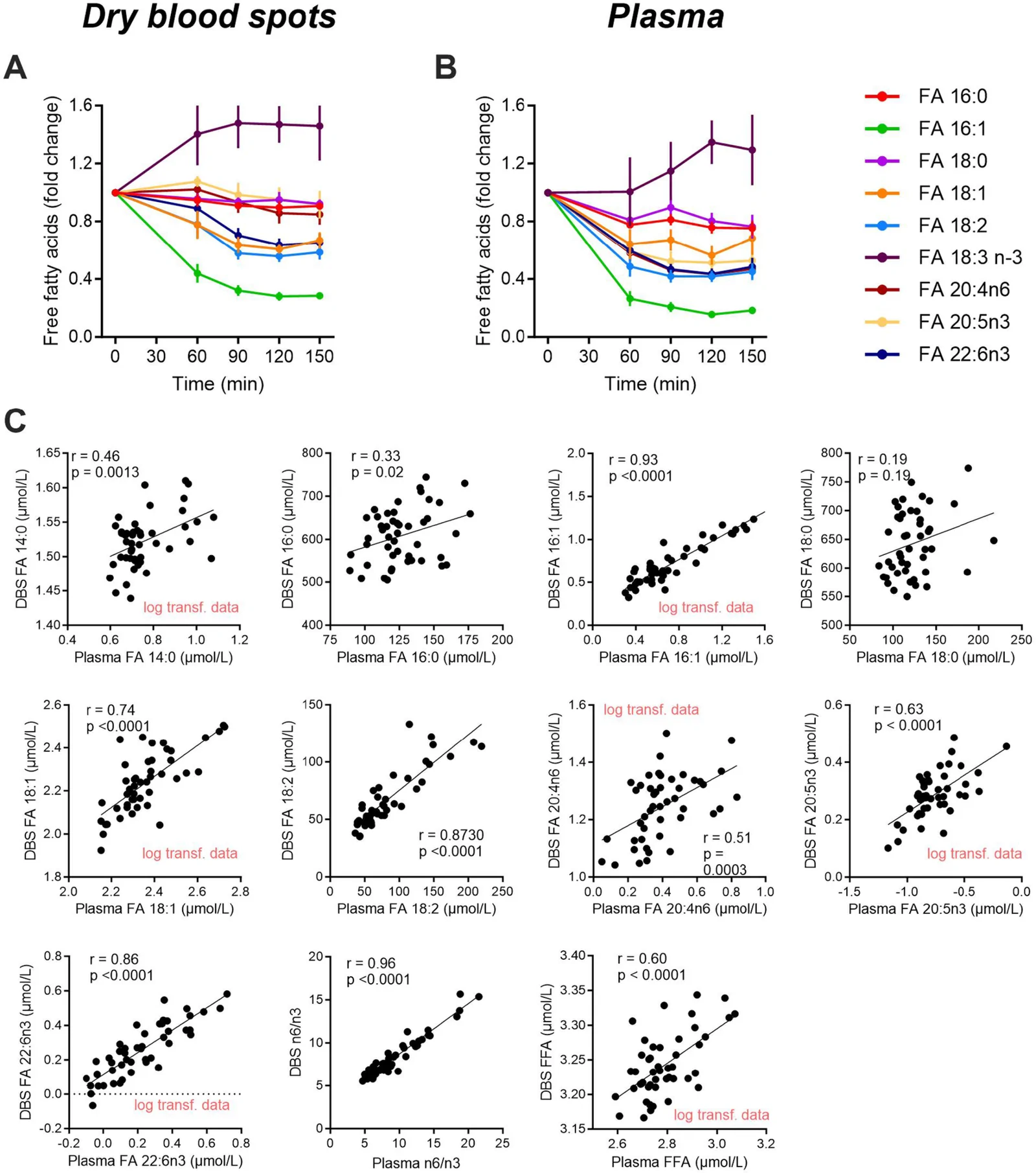
Changes in the levels of selected free fatty acids in DBS and plasma during the dietary challenge.** A**—Fold-changes of the most abundant/ clinically relevant free fatty acids in DBS during the dietary challenge. **B**—Fold-changes of the most abundant/ clinically relevant free fatty acids in plasma during the dietary challenge. **C**—Correlation between selected free fatty acids in plasma and DBS considering all time points of the dietary challenge. Data presented as mean ± standard error (*n* = 10)

The meal intake induced similar effects on the fatty acid profile in plasma and DBS, with reductions in FFA levels varying across fatty acid classes. The levels of monounsaturated fatty acids and omega-6 polyunsaturated fatty acids (n-6 PUFA) exhibited the largest decline during the postprandial period. Palmitoleic acid (FA 16:1) was the most responsive to meal intake in both matrices, with levels dropping by 74–86% at 90 min after meal intake (*q* = 0.043 in plasma and *q* = 0.038 in DBS) (Fig. 2). The nadir of FFA was observed at 90 min, when linoleic acid (FA 18:2n6), oleic acid (FA 18:1), and docosahexaenoic acid (DHA, FA22:6n3) also showed their lowest concentrations in plasma and DBS (*q* < 0.05). Noteworthy, arachidonic acid (ARA, FA 20:4n6) and eicosapentaenoic acid (EPA, FA 20:5n3) have their concentrations reduced by approximately 60% in plasma at 90 and 60 min (*q* = 0.0013 and 0.037, respectively), but do not show significant reductions in DBS (*q* = 0.111 and 0.638, respectively). Finally, the concentrations of palmitic acid (FA 16:0) and stearic acid (FA 18:0) in DBS were unaffected by meal intake, despite a trend towards decreased levels of these molecules in plasma (Fig. 2). The only fatty acid whose levels tended to increase in the postprandial period was α-linolenic acid (FA 18:3n3), which showed 38% increase in DBS and 37% increase in plasma, despite no statistical significance (Fig. 2).

Pearson correlation analysis confirms a positive correlation for the sum of FFA levels in both matrices (*r* = 0.60, *p* < 0.0001, Fig. 2). The correlation between FFA concentrations was higher for mono- and polyunsaturated fatty acids, including palmitoleic, oleic, linoleic, EPA, and DHA (*r* = 0.93, 0.74, 0.87, 0.63, and 0.86, respectively) and for the ratio of n-6/n-3 PUFA (*r* = 0.96, *p* < 0.0001). In contrast, saturated fatty acids such as palmitic and stearic showed comparatively weaker correlations (*r* = 0.33, and 0.19, respectively) (Fig. 2).

**Fig. 3:**
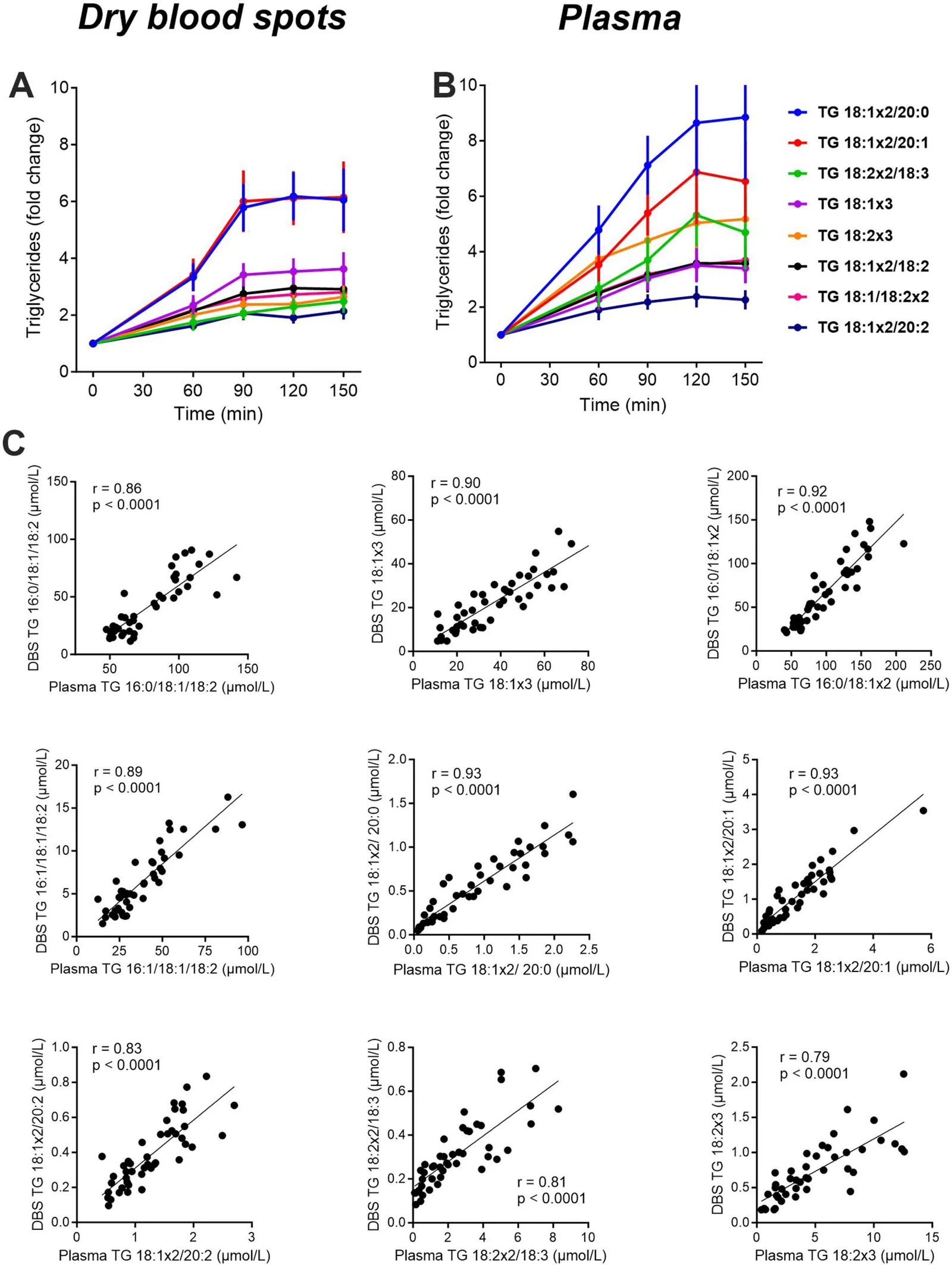
Changes in the levels of the triglycerides that most increased in DBS and plasma during the dietary challenge.** A**—Fold-changes of the triglycerides whose levels most increased in DBS during the dietary challenge. **B**—Fold-changes of the triglycerides whose levels most increased in plasma during the dietary challenge. **C**—Correlation between the levels of selected triglycerides in plasma and DBS considering all time points of the dietary challenge. Data presented as mean ± standard error (*n* = 10).

The TG profile was also found to be similar between capillary plasma and DBS samples. Among the 104 and 72 TG species quantified in plasma and DBS, respectively, 45 were shared between the two matrices. The kinetic pattern of TG appearance in circulation during the postprandial period was similar when comparing plasma and DBS samples. Among the 45 shared TG, eight that showed the greatest postprandial increase in plasma were likewise among those exhibiting the largest increases in DBS. Similar to FFA, TG concentrations in plasma and DBS showed a strong correlation (Fig. 3).

**Fig. 4:**
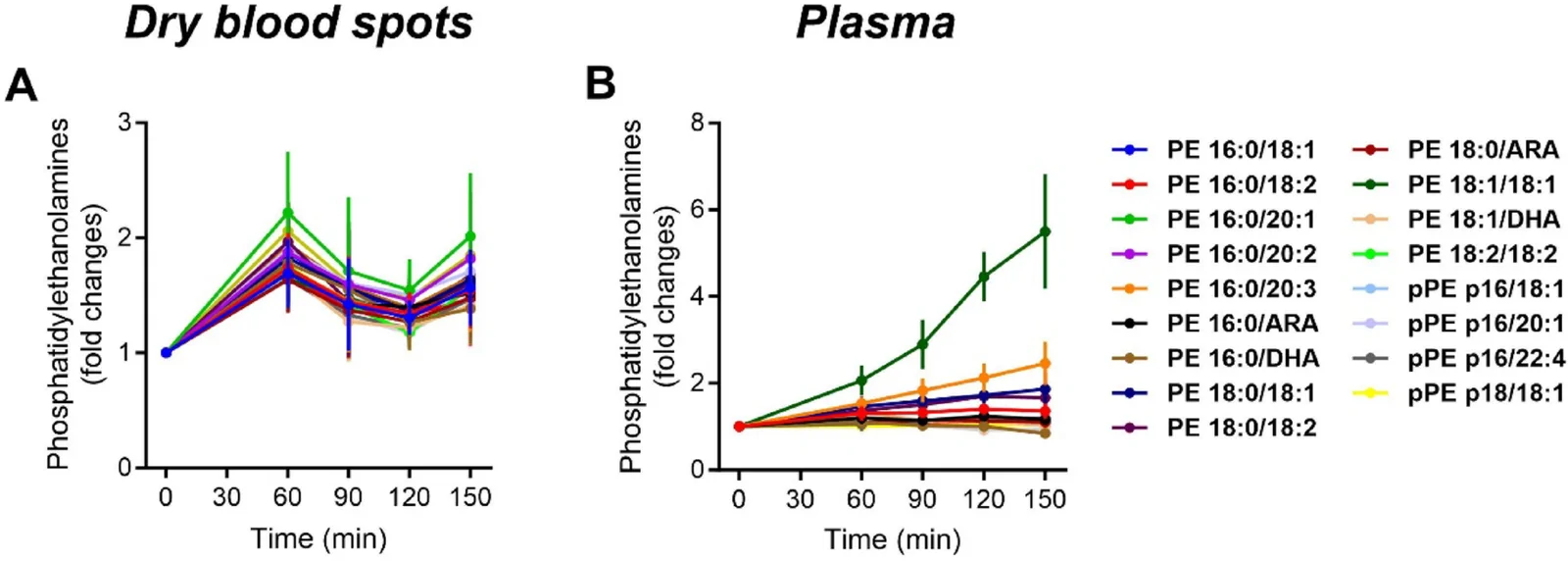
Phosphatidylethanolamines associated with discrimination of sampling time points during the dietary challenge in DBS.** A**—Phosphatidylethanolamine species identified among the most responsive lipids to the dietary challenge in DBS, expressed as fold-changes. **B**—Subset of phosphatidylcholines species shown in panel A that were also quantified in plasma, although not identified as discriminant variables (six PE species were not quantified in plasma). Data presented as mean ± standard error (*n* = 10)

In contrast to the above observations, the profiles of PE and pPE showed significant differences between capillary plasma and DBS. PE and pPE were among the most discriminating lipid species in DBS samples during the postprandial period (Table 2). Only a single PE species (PE 18:1/18:1) was also identified in plasma as a discriminant lipid among sampling points (Fig. 4 and Table 2). In DBS, the kinetic profile of pPE mirrored that of PE, displaying a bimodal pattern characterized by a two-fold increase within the first 60 min, followed by an approximate 40% decline by 120 min (Fig. 4).

**Fig. 5:**
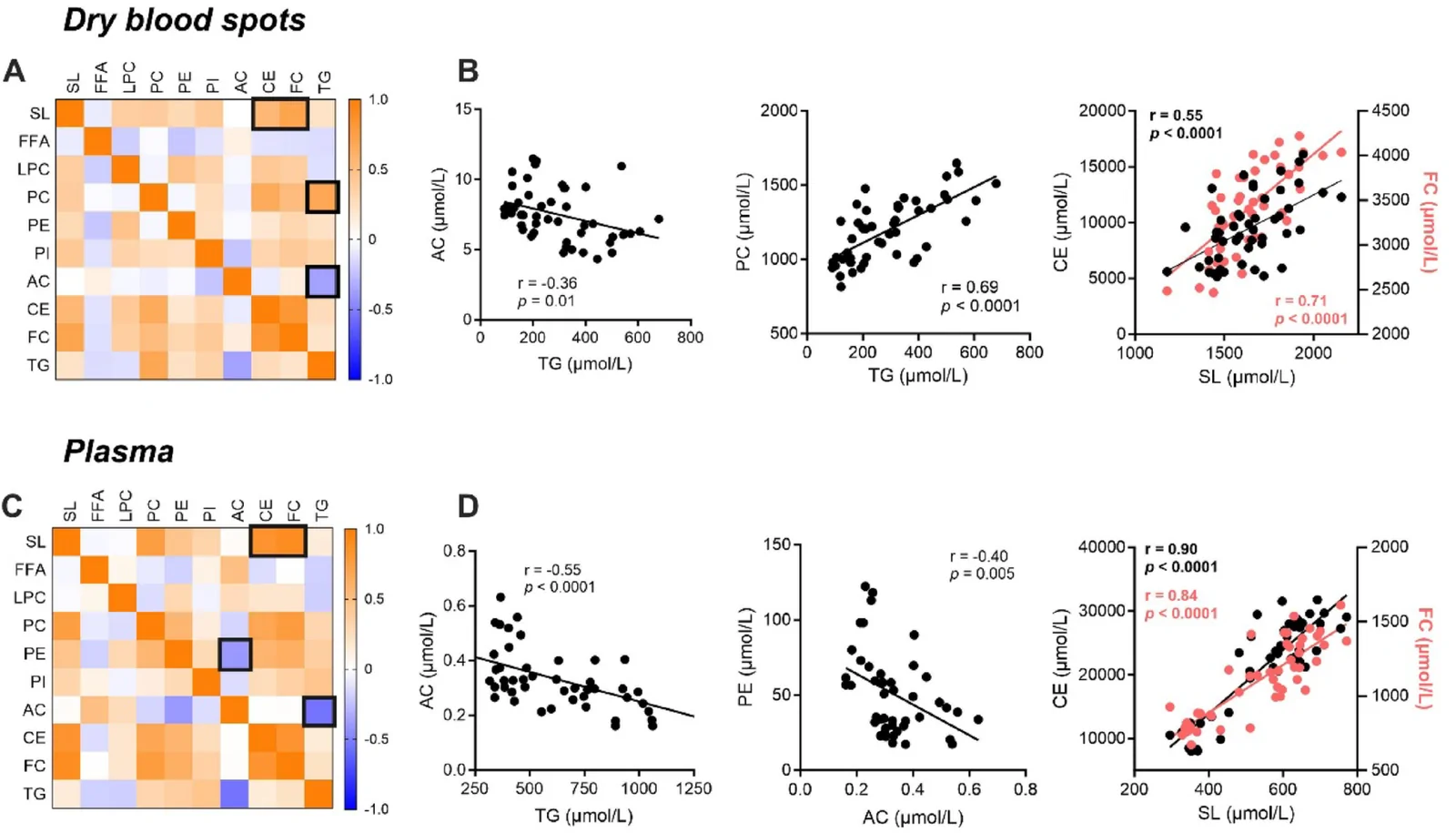
Correlations between lipid classes during the dietary challenge.** A**—Matrix of Pearson correlations between the levels of lipid classes in DBS (sum of all lipid species in each class). **B**—Correlations between selected lipid classes in DBS. **C**—Matrix of Pearson correlations between the levels of selected lipid classes in plasma (sum of all lipid species in each class). **D**—Correlations between lipid classes in plasma. FFA  = free fatty acids; CE  = cholesterol esters; FC  = free cholesterol; LPC  = lysophosphatidylcholines; PC = phosphatidylcholines; PE = phosphatidylethanolamines; PI = phosphatidylinositols; SL = sphingolipids; AC = acylcarnitines; TG = triglycerides

To provide an overview of the relationships among the most abundant lipid classes in capillary plasma and DBS, a correlation matrix was constructed using data from all sampling time points during the dietary challenge (Fig. 5). In DBS, AC and TG exhibited a significant negative correlation (*r* = − 0.36, *p* = 0.01), whereas PC and TG were positively correlated (*r* = 0.41, *p* = 0.004). Additionally, both CE and FC showed strong positive correlations with SL (*r* = 0.55, *p* < 0.0001 and *r* = 0.71, *p* < 0.0001, respectively) (Fig. 5b). In plasma, a similar pattern was observed, with AC and TG showing a significant negative correlation (*r* = − 0.55, *p* < 0.0001). Moreover, PE and AC were also inversely correlated (*r* = − 0.40, *p* = 0.005). Strong positive associations were identified between both CE and FC with SL also in plasma (*r* = 0.90, *p* < 0.0001 and *r* = 0.84, *p* < 0.0001, respectively) (Fig. 5d).

## Discussion

Despite the broad applicability of DBS in fields such as clinical biochemistry, infectious diseases, parasitology, and toxicology, there remains a lack of studies validating its use for assessing alterations in postprandial metabolism. Given the fluctuations in circulating lipid concentrations after the intake of a test meal, it could be questioned whether DBS can capture such changes, given that their basal lipid composition is intrinsically different from that of plasma. Our findings, however, demonstrate that the lipidome obtained from DBS is comparable to that of capillary plasma. Specifically, these results show that DBS captures the postprandial lipidome variations in terms of increased triglyceride concentration and decreased free fatty acid concentration after an energy-dense meal. Furthermore, we identified specific responses characterized by the increased concentration of phosphatidylethanolamines in DBS.

Circulating lipid levels are markedly affected by the consumption of a high-fat meal, with pronounced postprandial changes observed in plasma levels [12]. Given that most individuals with safe access to food usually consume three fat-containing meals per day, it can be assumed that they spend the majority of their waking hours in a postprandial state, characterized by an altered plasma lipidome. Here, we report that the dynamic postprandial lipidome typically monitored in plasma is also replicated in DBS samples. Previous studies have contributed to the discovery of novel biomarkers linked to postprandial lipid metabolism, which may be useful for the diagnosis, prevention, and management of metabolic disorders and chronic diseases such as cardiovascular disease and nonalcoholic fatty liver disease [13, 14]. The major contribution of this study was to demonstrate that, although DBS samples are rich in phospholipids due to hematocrit, their use as a sampling strategy during dietary challenges can capture changes in lipid levels comparable to those observed in capillary plasma. It is important to note that capillary blood represents a mixture of arterial and venous blood, as well as interstitial fluid, which may result in compositional differences relative to conventional venous blood. This should be considered when interpreting lipidomic data and comparing findings across studies using different sampling strategies.

Lipidomics-based postprandial studies are increasingly recognized for their potential to uncover dynamic metabolic traits that are not detectable in the fasting state. By capturing these transient responses, such studies may contribute to developing standardized methodologies and biomarkers for use in clinical practice, enabling the timely and accurate identification of individuals at increased risk for chronic diseases [15]. Notably, in this study, DBS samples mirrored the decline in circulating FFA levels observed in plasma samples. The elevated circulating FFA levels characteristic of an overnight fast result from lipolysis in adipose tissue and other tissues, a process suppressed by the postprandial rise in insulin, which drives FFA levels down [16–18]. Fatty acids and products of their metabolism have previously been described among the metabolites whose plasma concentrations undergo significant alterations during a dietary challenge [19]. These plasma concentrations decrease markedly within the first few hours following the challenge and begin to rise again after approximately two hours [20, 21]. Changes in circulating fatty acid levels between fasting to postprandial states have been associated with insulin sensitivity [22–24]. Palmitoleic acid exhibited the most marked postprandial decrease in both DBS and plasma. Similarly, linoleic acid levels were also dramatically decreased following food intake. A previous study reported that linoleate is preferentially mobilized during lipolysis by hormone-sensitive lipase (HSL) in the adipose tissue, compared to other polyunsaturated fatty acids [25]. While the selectivity of HSL toward highly unsaturated fatty acids remains to be fully elucidated, some evidence suggests that carbon chain length exerts a greater influence on HSL specificity than the degree of unsaturation [26]. Conversely, other findings indicate that the degree of fatty acid unsaturation has a more pronounced effect on hydrolysis rates than chain length [27].

Among the FFA that differentiated DBS from plasma samples, eicosapentaenoic acid (EPA, FA 20:5n3) and arachidonic acid (ARA, FA 20:4n6,) were particularly notable. In DBS samples, EPA and ARA levels remained relatively unaltered during the postprandial period, whereas in plasma, both exhibited the expected decline. This observation may be partially explained by the differential cellular composition of DBS, which includes cellular components such as erythrocytes, leukocytes, and platelets—cell types in which FA are integral components of membrane phospholipids. ARA, in particular, is a major polyunsaturated fatty acid in cellular membranes, conferring structural fluidity and participating in critical membrane-associated processes such as permeability regulation, capping, endocytosis, and immune signaling [28]. Thus, their relative stability in DBS does not directly reflect circulating FFA dynamics, but rather the contribution of membrane-bound fatty acids that are not subject to rapid postprandial fluctuations. In contrast, plasma FFA more accurately represents the dynamic circulating pool regulated by lipolysis and insulin action.

On the other hand, evidence indicates that EPA displays distinct kinetic profiles across biological compartments. While it is rapidly cleared from the plasma FFA pool within hours, EPA persists in plasma phospholipids and remains incorporated into erythrocyte membranes for extended periods. Accordingly, measurement of EPA and docosahexaenoic acid (DHA) in erythrocyte membrane phospholipids is considered a more robust and temporally stable biomarker with lower variability than measurement in plasma, due to the limited exchange between plasma and cellular compartments [29]. Furthermore, the selective postprandial increase in α-linolenic acid (FA 18:3n3) likely reflects its high abundance in canola oil [30] and low fasting circulating levels. This response underscores the ability of DBS sampling to detect acute diet-induced fluctuations in fatty acid composition consistent with plasma measurements.

The findings of our study indicate that TG alterations after a meal are effectively captured using DBS sampling, indicating the potential of this approach as a sustainable tool for monitoring changes in lipid metabolism during the postprandial period. Following food intake, circulating triglyceride levels typically rise, peaking within 3–4 h after the meal, and gradually return to baseline within 6–8 h after the meal [31]. In some cases, TG levels may remain elevated above baseline for up to 12 h, depending on factors such as meal composition and individual metabolic variability [32]. Meal composition is a primary determinant of the magnitude of the postprandial TG response, with high-fat meals eliciting a more pronounced lipemic excursion compared with low-fat meals. For instance, Keirns et al. [33] observed a 236% rise in TG levels following a high-fat meal, indicating a direct relationship between dietary lipid content and postprandial lipemia. Importantly, TG concentrations measured between 2 and 4 h postprandially have been shown to display a stronger association with cardiovascular disease risk than fasting TG measurements [34]. In our study, the increase in total TG was milder, likely due to the use of canola oil, which is rich in monounsaturated fats and is known to induce a lower postprandial rise in triglyceride levels [35, 36]. Additionally, the relatively short sampling period (150 min) may have contributed to the modest TG response, as peak concentrations are typically reported between 3 and 4 h after meal intake. The present study focused on the early to mid-postprandial phase, characterized by rapid, insulin-mediated metabolic adjustments—often peaking around 30 min after ingestion of a mixed meal [36, 37]—including suppression of free fatty acids and the onset of triglyceride elevation. This simplified dietary challenge that combined minimally invasive blood sampling, and a rather short dietary challenge was enough to observe key changes in lipid metabolism. Further studies incorporating longer postprandial sampling periods are warranted to provide a more comprehensive characterization of lipid kinetics using DBS.

Due to their chemical diversity, triglycerides represented the most abundant lipid class (in terms of number of species) in both DBS and plasma, accounting for more than 20% and 30% of total lipid species, respectively. As expected, postprandial TG levels in both matrices exhibited a marked increase following consumption of the mixed liquid meal. Detailed analysis revealed that TG species containing acyl chains with 15–22 carbon atoms and up to four double bonds were responsive postprandially in plasma, DBS, or both. Likely due to the high oleic acid content of the canola oil (~ 60%) used in the test meal, TG species containing oleic acid were among those that displayed the most pronounced postprandial responses.

Snowden et al. [35] reported that, although plasma samples typically yield a greater number of detectable lipid species than DBS, a substantial proportion of lipids identified in DBS are also present in plasma. These authors identified a panel of lipids that could predict the concentration of ‘clinically relevant lipids’, with correlations with measured triglycerides, HDL, LDL and total cholesterol of 0.92, 0.74, 0.58 and 0.42 respectively, in plasma samples, whereas DBS samples display correlations of 0.84, 0.59, 0.561 and 0.57 [38]. Furthermore, in a randomized population-based study, comparable triglyceride levels were observed between venous and DBS samples, with a significant correlation between the two sampling methods [39]. While these studies validated DBS as a surrogate for plasma in the fasting state, our findings extend this validity to samples collected after a high-fat meal, when significant changes in blood lipidome is expected.

The levels and metabolism of triglycerides in the bloodstream can influence circulating phospholipid levels. Plasma lipoproteins, which serve as the primary carriers of lipid in circulation, originate predominantly from the liver and the intestine. Both organs produce triglyceride-rich lipoproteins as part of lipid transport processes. The liver synthesizes and secretes very-low-density lipoproteins (VLDL), while the intestine secretes chylomicrons following dietary fat absorption. Although these lipoproteins are characterized by their high triglyceride content, they also contain substantial amounts of phospholipids, as well as free and esterified cholesterol [40]. Notably, the liver maintains continuous phospholipid synthesis to support the formation and secretion of lipid droplets and VLDL particles [41]. Among phospholipids, PC plays a central role in hepatic VLDL assembly and secretion. PC is required for stabilizing the surface monolayer of nascent VLDL particles, and impaired PC availability is known to hinder VLDL secretion, leading to triglyceride accumulation in the liver [42, 43]. Evidence from a genome-wide association study has demonstrated that distinct triglyceride species are positively correlated with several phospholipid species, suggesting the involvement of shared biosynthetic pathways or coordinated regulatory mechanisms governing the metabolism of both lipid classes [44].

These observations highlight the integrated roles of triglycerides and phospholipids in lipid transport, metabolic regulation, and cardiovascular disease risk. However, important matrix-specific differences must be considered when interpreting DBS data. Unlike plasma, DBS reflects whole blood and therefore include cellular components such as leukocytes and platelets. Given that a hallmark of the postprandial state is an increase in the number of circulating leucocytes [36, 45, 46], the marked responsiveness of both phosphatidylethanolamine and their plasmalogen forms in DBS may partly reflect their abundance in immune cells [47] rather than changes exclusively occurring in circulating lipoproteins. Consequently, while DBS effectively captures postprandial dynamics of lipids linked to lipoprotein metabolism (e.g., triglycerides and free fatty acids), it may also incorporate signals derived from cellular lipid contributions. Thus, DBS and plasma provide complementary rather than interchangeable information, with DBS being particularly suitable for investigating selected lipid classes and integrated systemic responses, whereas plasma remains preferable when the objective is to assess lipoprotein-specific lipid metabolism.

## Conclusion

This study demonstrates that DBS capture changes in the postprandial circulating lipidome, yielding metabolic responses that reflect those observed in capillary plasma. There was an overlap in lipid species between matrices, with variations arising mainly in intensity rather than presence. Free fatty acids and triglycerides emerged as the most dynamically regulated lipid classes following meal intake, exhibiting similar kinetic patterns in both matrices. DBS-specific lipidomic signatures were also observed, particularly for glycerophospholipids and phosphatidylethanolamines, likely reflecting the contribution of cellular components in whole blood. These findings support the use of DBS as a complementary, minimally invasive approach for assessing postprandial lipid metabolism, especially for selected lipid classes such as triglycerides and free fatty acids. Rather than a direct substitute for plasma, DBS should be interpreted in a context-dependent manner, considering the lipid species and the biological question of interest. Future studies should further investigate matrix-specific lipid signatures to enhance the clinical and research utility of DBS technology.

## Supplementary Information

Below is the link to the electronic supplementary material.

Supplementary Material 1

Supplementary Material 2

Supplementary Material 3

Figure S2. Partial least square discriminant analysis (PLS-DA) model plots. A - Score plot from the PLS-DA model built using data from plasma samples. B - Loadings plot from the PLS-DA model built using data from plasma samples. C - Permutation plot to assess que quality of the PLS-DA model built using data from plasma samples. D - Score plot from the PLS-DA model built using data from DBS samples. B - Loadings plot from the PLS-DA model built using data from DBS samples. C - Permutation plot to assess que quality of the PLS-DA model built using data from DBS samples.

## Data Availability

No datasets were generated or analysed during the current study.
