# Supplementary material for: Comparative analysis of postprandial changes in the lipidome of capillary plasma and dry blood spots of young women
Source: Eur J Nutr. 2026 Oct 7;65(8):266. doi: 10.1007/s00394-026-04119-z (PMC13645943; doi:10.1007/s00394-026-04119-z)
Supplement: Supplementary file 1 [file 394_2026_4119_MOESM2_ESM.pdf]

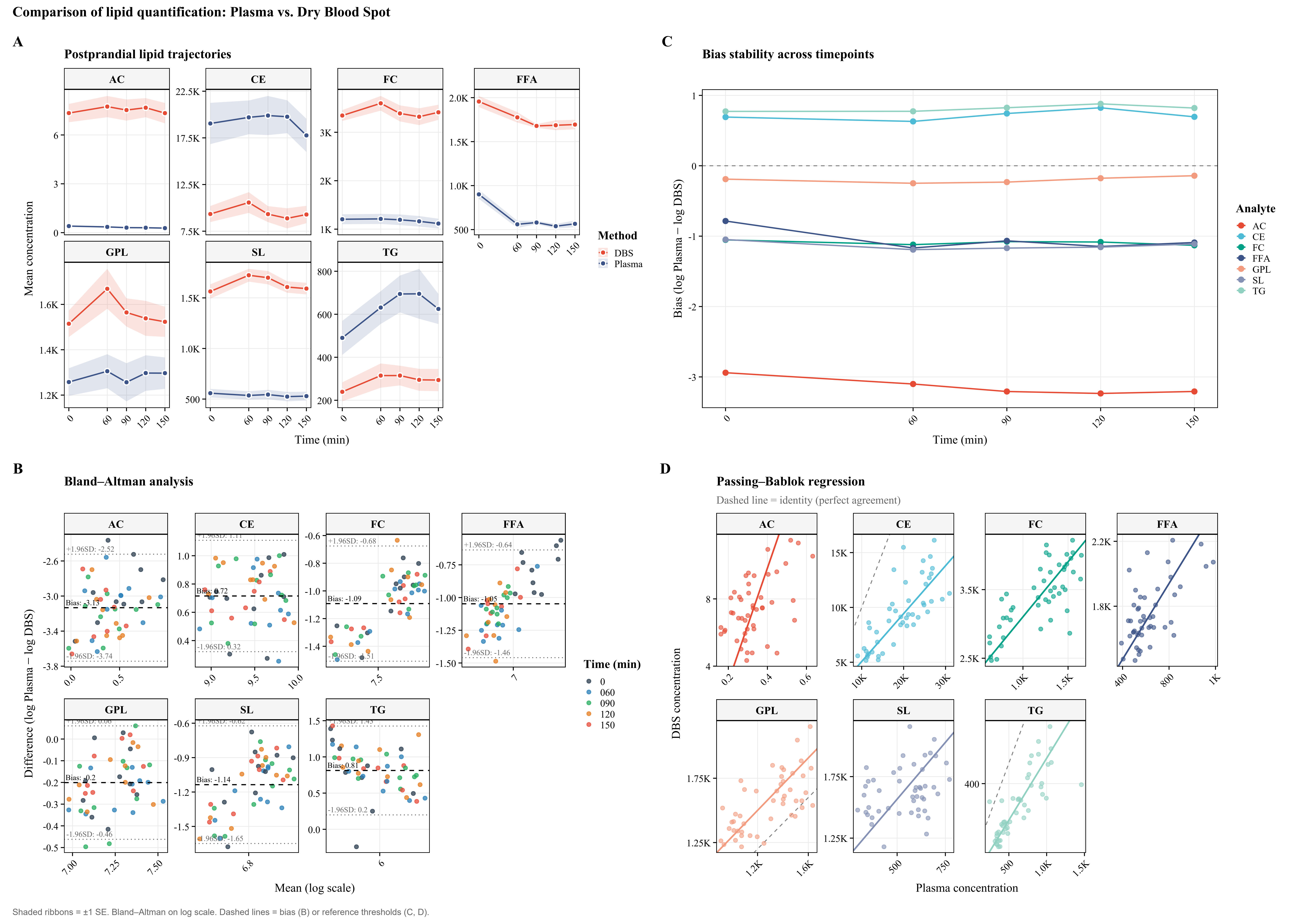

Comparison of lipid quantification: Plasma vs. Dry Blood Spot
A
Postprandial lipid trajectories
AC
CE
FC
FFA
GPL
SL
TG
Mean concentration
Time (min)
Method
DBS
Plasma
C
Bias stability across timepoints
Bias (log Plasma − log DBS)
Time (min)
Analyte
B
Bland–Altman analysis
Difference (log Plasma − log DBS)
Mean (log scale)
Time (min)
D
Passing–Bablok regression
Dashed line = identity (perfect agreement)
DBS concentration
Plasma concentration
Shaded ribbons = ±1 SE. Bland–Altman on log scale. Dashed lines = bias (B) or reference thresholds (C, D).
