# Supplementary material for: Comparative analysis of postprandial changes in the lipidome of capillary plasma and dry blood spots of young women
Source: Eur J Nutr. 2026 Oct 7;65(8):266. doi: 10.1007/s00394-026-04119-z (PMC13645943; doi:10.1007/s00394-026-04119-z)
Supplement: Supplementary file 3 [file 394_2026_4119_MOESM4_ESM.pdf]

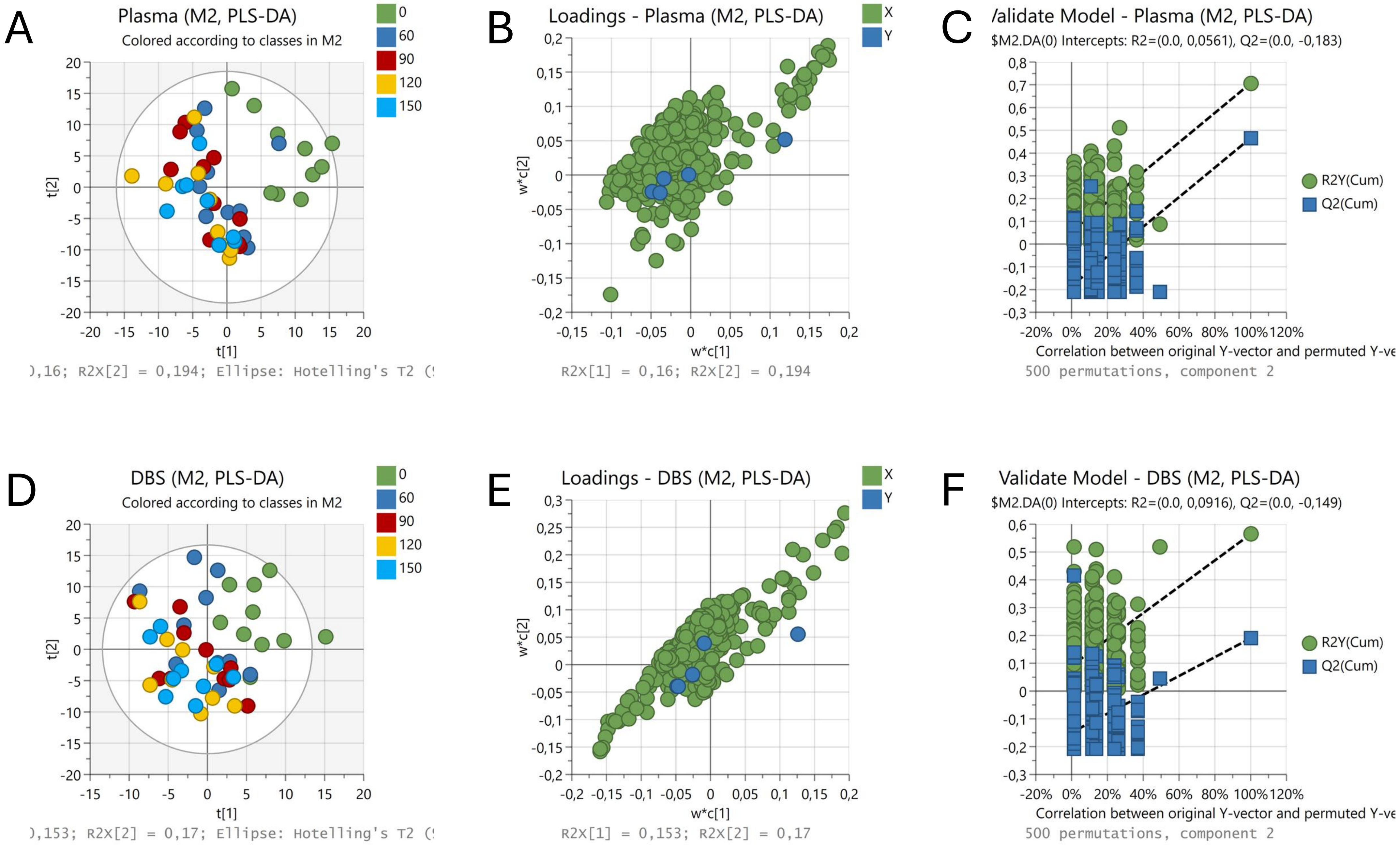
A
Plasma (M2, PLS-DA)
Colored according to classes in M2
0
60
90
120
150
t[2]
t[1]
),16; R2X[2] = 0,194; Ellipse: Hotelling's T2 (
B
Loadings - Plasma (M2, PLS-DA)
X
Y
w*c[2]
w*c[1]
R2X[1] = 0,16; R2X[2] = 0,194
C
/alidate Model - Plasma (M2, PLS-DA)
$M2.DA(0) Intercepts: R2=(0.0, 0,0561), Q2=(0.0, -0,183)
R2Y(Cum)
Q2(Cum)
Correlation between original Y-vector and permuted Y-ve
500 permutations, component 2
D
DBS (M2, PLS-DA)
Colored according to classes in M2
0
60
90
120
150
t[2]
t[1]
),153; R2X[2] = 0,17; Ellipse: Hotelling's T2 (
E
Loadings - DBS (M2, PLS-DA)
X
Y
w*c[2]
w*c[1]
R2X[1] = 0,153; R2X[2] = 0,17
F
Validate Model - DBS (M2, PLS-DA)
$M2.DA(0) Intercepts: R2=(0.0, 0,0916), Q2=(0.0, -0,149)
R2Y(Cum)
Q2(Cum)
Correlation between original Y-vector and permuted Y-ve
500 permutations, component 2
