# Supplementary material for: Comparative analysis of postprandial changes in the lipidome of capillary plasma and dry blood spots of young women
Source: Eur J Nutr. 2026 Oct 7;65(8):266. doi: 10.1007/s00394-026-04119-z (PMC13645943; doi:10.1007/s00394-026-04119-z)
Supplement: Supplementary file 4 [file 394_2026_4119_MOESM1_ESM.docx]

**Supplemental Table S1. Internal standards used in the lipidomic analysis.**

| **Positive mode mix** | | **Negative mode mix** | |
| --- | --- | --- | --- |
| *Lipid* | *Concentration (µg/mL)* | *Lipid* | *Concentration (µg/mL)* |
| AC (12:0) | 20 | Cer (d18:1/17:0) | 10 |
| Cholesterol d6 | 100 | CL (14:0x4) | 20 |
| CE (16:0) d7 | 20 | FFA (13:0) | 20 |
| CE (17:0) | 100 | 2HexCer (d18:1/17:0) | 5 |
| CoQ 6 | 2 | HexCer (d18:1/17:0) | 5 |
| DG (14:0x2) | 50 | LPC (17:1) | 10 |
| TG (14:0x3) | 50 | LPC (19:0) | 25 |
| TG (17:0x3) | 2,8 | LPE (17:1) | 5 |
|  |  | PA (14:0/14:0) | 20 |
|  |  | PC (17:0/17:0) | 25 |
|  |  | PC (21:0/21:0) | 20 |
|  |  | PE (14:0/14:0) | 20 |
|  |  | PE (17:0/17:0) | 25 |
|  |  | PG (14:0/14:0) | 20 |
|  |  | PG (17:0/17:0) | 25 |
|  |  | PS (14:0/14:0) | 25 |
|  |  | PS (17:0/17:0) | 25 |
|  |  | SM (d18:1/17:0) | 5 |
|  |  | Sulfatide (d18:1/17:0) | 1 |
